# I determine my learning path, or not? A study of different learner control conditions in online video-based learning

**DOI:** 10.3389/fpsyg.2022.973758

**Published:** 2022-09-08

**Authors:** Lu Li, Xinghua Wang, Matthew P. Wallace

**Affiliations:** ^1^School of Literature and Journalism, Qingdao University, Qingdao, China; ^2^Normal College, Qingdao University, Qingdao, China; ^3^Faculty of Arts and Humanities, University of Macau, Macau, Macau SAR, China

**Keywords:** learner control, learning path, video-based learning, transactional distance, online

## Abstract

Learner control is an important instructional design in video-based learning. This study assessed two conditions: a full learner control where learners direct their learning path, and a hybrid learner control where learners follow the instructor-set path but still enjoy certain aspects of control. Two groups of university students participated in this study by learning statistics through online video courses. The findings show that the full learner control condition attained higher learning performance than the hybrid learner control condition, but spent more time than the latter. The full learner control condition scored higher than the hybrid condition in the difficult sections of video-based learning; but no significant difference was found in the easy section. There was a significant difference between the two conditions in learning agency, but no differences in cognitive load and affective and cognitive engagement. Hierarchical regression analysis indicated differences between the full and the hybrid learner control conditions in the factors predicting overall scores. The findings carry important contributions and implications for the research and practice of instructional designs in online video-based learning such as MOOCs.

## Introduction

Extant research (e.g., Mihalca et al., 2017; Biard et al., 2018; Chang et al., 2021) has been arguing around self-directed learning path or instructor/system-directed learning path in online video-based learning, such as massive online open courses (MOOCs) and small private online courses (SPOCs) widely used during the COVID-19. Such argument is essentially a question about how to optimize the presentation of instructional videos to facilitate student learning due to the transient information flow characterized by online videos and the cognitive cost of processing the information (Biard et al., 2018; Schroeder et al., 2020). This argument is further translated into the issue of learner control (Biard et al., 2018; Bétrancourt and Benetos, 2018). Learner control in this study refers to learners’ agentic power over the interaction with instructional videos, such as determining the sequencing and pacing of information presentation in instructional videos, and allows learners to allocate their cognitive resources based on their needs and capacities (Scheiter and Gerjets, 2007; Bétrancourt and Benetos, 2018). It is situated in a continuum from no learner control (i.e., system or instructor control; used interchangeably in this study) to full learner control (Schroeder et al., 2020).

With video-based learning gaining momentum in recent years, particularly during the pandemic (e.g., Capon-Sieber et al., 2021; García-Morales et al., 2021), it is of paramount importance to identify which type of learner control work for students, that is, letting the students determine their learning path or not. This study aims to fill this gap by conducting a quasi-experimental design following the theory of transactional distance. However, as pure instructor/system-control is rarely seen in current online learning considering that the idea of student-centered learning has been widely accepted and student agency is highly encouraged, this study will not examine this design.

## Prior studies on learner control in video-based learning

Prior studies have been conducted on learner control vs. system/instructor control in both procedural and conceptual learning, which are two ways of acquiring knowledge (Adeleke, 2007; Pozo et al., 2021), using instructional videos. However, mixed findings were identified related to the effect of two types of controls on student learning. It remains unclear what kind of learner control works best (Tabbers and de Koeijer, 2010; Biard et al., 2018).

As for procedural learning (i.e., learning through performing a series of actions; Biard et al., 2018), for instance, Schwan and Riempp (2004) investigated how participants learned to tie nautical knots by using interactive and non-interactive videos. In the interactive condition, the participants could control their learning pace by using interactive features such as pausing, replaying, and controlling the speed of presentation. They could practice tying knots at any time by pausing the video. In the non-interactive condition, the participants had to watch the video from beginning to end at normal speed without stopping. The participants had to wait until the end of the video before they could practice tying knots. Results showed that the participants heavily used the interactive features to learn to tie nautical knots, particularly the difficult knots, and that their learning effectiveness was higher than the participants in the non-interactive condition. Biard et al. (2018) examined how the features of pausing and segmentation of an instructional video affected the learning of a medical procedure. They hypothesized that the students would make little use of the pause button as they would not know when to stop the video, and that learner-paced pausing used together with system-paced segmentation could improve procedural learning. Students were divided into three groups: non-interactive video where the students could not pause the video; interactive video where the students could pause the video anytime they wanted (i.e., learner-paced control); and segmented interactive video where the students could pause but only at segments set by the system (i.e., system-paced control). Results indicated that the system-paced control condition outperformed other conditions in procedural learning, but there was no significant difference in recall tests among the three conditions.

As for conceptual learning, defined by Adeleke (2007) as learning through acquiring knowledge of conceptions and principles, Merkt et al. (2018) examined whether pauses benefited student learning in a long instructional video (lasting 773 s) about “Acoustic Oscillations.” They restructured the long video into four conditions (a continuous video without pauses or structural markers, a video with structural markers at meaningful breakpoints, a video with pauses at meaningful breakpoints, and a video with pauses at meaningless breakpoints) which were tested in two experiments. However, they did not identify the beneficial effects of pauses on learning the instructional video. Schroeder et al. (2020) investigated the effects of three conditions (system-paced, learner-paced, and learner-attenuated system paced [LASP]) on learning the formation of lightning which was adapted from Moreno and Mayer (1999). In the system-paced condition, the participants had no control over the video and could watch it once. In the learner-paced condition, the video was segmented into 16 clips with each lasting from 4 to 11 s. The participants could review the clips but could not revisit the clips after moving forward. In the LASP condition, the participants had full control over the video, for instance, pausing, rewinding, and skipping content. Results showed that the participants in the system-paced condition had the lowest performance. Those in the learner-paced condition and the LASP achieved similar performance.

Overall, the aforementioned and other similar studies provide commendable examples for the experimentation of varying designs of learner control. However, besides the mixed findings of learner control, there exist several other issues that call for further investigation. For instance, they mostly focused on student learning in a single video. Little is known regarding student learning in a series of instructional videos with different difficulty levels, which are quite common in practice. Some learning tasks were not authentic, for example, presenting the formation of lightning, which was quite basic, for university students (e.g., Tabbers and de Koeijer, 2010; Schroeder et al., 2020). Consequently, research results from the participants’ responses might be subject to caution (Hummel et al., 2021). Moreover, there is limited knowledge about the effectiveness of full learner control over the entire path of video-based learning. Full learner control involves the control over not only the pace of a specific video but also the sequence of a series of instructional videos that are arranged following an easy-to-complex principle and together form a (mini) course (Schroeder et al., 2020).

Another more critical issue may be the questionable premise of some studies. For example, the system-paced videos where learners have no control over at all in Schroeder et al. (2020) are not often encountered and used in actual learning. In a continuum from no learner control to full learner control, there exists another control condition called “Hybrid learner control” in this study. In the hybrid learner control condition, learners can control the display and pace of a specific video but have to follow the presentation sequence of instructional videos predetermined by their instructors. For instance, in some flipped classrooms, learners have to go through all videos one by one, the path of which is set by their instructors, before attending face-to-face sessions. Such design is to ensure that the students do not go out of their way to skip certain course content and are well equipped with the knowledge needed for subsequent learning activities. But the learners can still control the display of each video. Thus, this type of video-based learning is neither purely system-controlled nor purely learner-controlled, but combine both.

Therefore, in this study, we would not examine the pure system-controlled videos as prior studies did. Nor would we test learner control in a single video. Instead, we compared the full learner control condition (FLC in short) with the hybrid learner control condition (HLC in short) which involved both learner control and instructor control in a mini course involving four videos with each lasting 10–12 min so as to reveal what kind of learner control might work better. The learning task in this study was related to university-level statistics learning, which was authentic to the university student participants and is increasingly offered in online formats in recent years (Huang and Mayer, 2019).

In the present study, we primarily used the theory of transactional distance as the theoretical framework. Moreover, to better explain how students managed the information processing in video-based learning, we also consulted the theory of cognitive load and the cognitive theory of multimedia learning.

## Theoretical foundation

Transactional distance plays an important role in digital learning as it is related to the psychological and communication space to be crossed, which is also a space potentially causing misunderstanding between students and objects of digital learning environments such as course content, people, and technology (Moore, 1993; Jung et al., 2019; She et al., 2021). It is strongly related to students’ engagement in and satisfaction with digital learning and consequently their academic success (Yılmaz and Keser, 2017; Weidlich and Bastiaens, 2018).

Transactional distance is a pedagogical concept rather than a geographic phenomenon (Moore, 1993). Among the different forms of interaction (e.g., student-student and student-instructor interactions) in digital or blended learning, the transactional interaction between students and course content may be the most critical form and has a greater effect on learning performance than other forms (Ekwunife-Orakwue and Teng, 2014). The student-content interaction is influenced not only by the subject matter but also by the instructional design of the course (Moore, 1993; Yılmaz and Keser, 2017). As such, the transactional distance associated with the student-content interaction can be decreased or overcome by effective and deliberate instructional designs and can also be increased if instructional designs are not planned well (Chen, 2001; Ekwunife-Orakwue and Teng, 2014).

The variations of learner control constitute an important form of instructional design (Jung et al., 2019). The learner control over the pace of video-based learning determines the interaction between learners and course content as well as the learners’ agentic behaviors, thereby affecting the transactional distance between the learners and course content (Moore, 1993; Jung et al., 2019). Therefore, this study considers that the transactional distance theory provides an effective framework to explain how students with different types of learner control interact with instructional videos and how different interactions cause transactional distance, which further leads to different academic performances.

In addition, according to the cognitive theory of multimedia learning (Mayer, 2005) as well as the cognitive load theory (Sweller et al., 1998), the continuous and synchronous flow of visual and auditory information generated by a video can lead to a heavy cognitive load. If learners cannot control the presentation pace of information in a video, which is fed to the learners quickly, they tend to experience essential overload where the cognitive processing requirements of educational resources exceed the learners’ cognitive capacities (Sweller et al., 1998; Mayer, 2005). For this reason, it is critical to examine how learners can control their pacing in video-based learning to avoid essential overload as well as to enhance their learning performance.

In line with the abovementioned analysis, this study aimed to answer the following research question: How do different types of learner control work for students in online video-based learning?

## Methodology

### Participants and research context

A total of 86 university students from a department of Literature and Journalism took part in this study. They were randomly assigned to the full learner control condition (FLC) with 44 students (38 females and 6 males) and the hybrid learner control condition (HLC) with 42 students (31 females and 11 males). They were aged between 18 and 20 years. This study was conducted with ethical clearance from the university and obtained informed consent from the participants.

All the participants did not take any statistics course before. However, to verify their prior knowledge of statistics, we conducted a pretest 1 day before the experiment using a standard test of statistics which comprised 20 items with 100 points in total. The results showed no significant difference between FLC (*M* = 60.89; *SE* = 2.27) and HLC (*M* = 59.40; *SE* = 1.47), *t*(84) = 0.54, *p* = 0.59.

### Research designs

The participants were required to finish a mini course and associated tests which were hosted on a website. The course in this study comprised four instructional videos: Central tendency, Dispersion, Random variables and distributions, and Probability density function, each lasting from 10 min 10 s to 12 min 32 s. They were organized following a structured sequence based on a simple-to-complex principle, which could increase students’ interest and improve their learning performance (Hew et al., 2018; Jung et al., 2019). The first two videos were easier and simpler than the latter two videos. In addition, the first two videos made up the knowledge base for the latter two videos. To better understand the content in the latter two videos, the students needed to master the knowledge in the first two videos. Subsequent to each video, there was a test consisting of two parts: retention test and transfer test, each with five items. Each item was assigned one point. The total score for the four videos was 40 points. The higher the score, the better the academic performance.

All students in FLC and HLC were briefed about the structure of the online course at the onset of this study. This was to make them aware of the content and workload associated with each video and test so as to inform their decision-making of the video-based learning (Schroeder et al., 2020). The informed learner control was also an important feature distinguishing this study from many prior studies and constituted an aspect of authentic tasks in practice.

**FIGURE 1:**
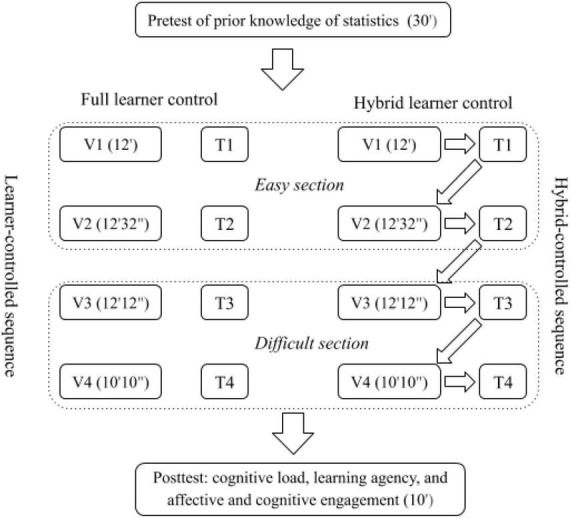
Procedure of this study. V, Video; T, Test; ’Minute; “Second.

The students in FLC determined the learning path by themselves. They had full control over the entire learning pace. Specifically, they could determine which video to watch firstly, whether to skip any video or not, whether to watch the videos first or do the tests first, and how to watch each video (e.g., at a slower or faster pace, or skip certain content). In contrast, the students in HLC had to follow the learning path set by their instructor. They had to finish the instructional videos and the associated tests one by one and could not skip around. But they still could pause, accelerate, slow down, and replay the very instructional video that they were watching. After finishing all videos and tests, they could go back to watch any video they liked. Overall, the main difference between FLC and HLC is the learning path. That is, FLC was a learner-controlled path while HLC was a hybrid-controlled path. Figure 1 visualizes the procedure of this study.

### Measures

To answer the research questions, multiple data sources were collected, including retention test, transfer test, and time-on-task. Survey data were also collected, including cognitive load, learning agency, and affective and cognitive engagement. The items of the survey (see Supplementary Appendix A) were rated on a five-point Likert scale, with 1 indicating “strongly disagree” and 5 “strongly agree.” In addition, focus group interviews were carried out at the end of this study.

#### Retention test, transfer test, and score calculation

The retention test following each instructional video covered the key content presented in the video and examined the participants’ comprehension of the statistics knowledge. The transfer test tapped on the content that was not directly discussed in the videos, but that could be answered by inferring from the key content covered in the videos. Both the retention and transfer tests consisted of multiple-choice questions (see Supplementary Appendix B for sample questions). They were developed by the instructor of this course and a statistician. Many items in both tests were related to calculation and reasoning. The total retention and transfer test scores for FLC and HLC were computed separately. The overall score for each design was computed by adding up the scores of the retention and transfer tests. The scores of the easy and difficult sections were calculated separately for FLC and HLC by adding up the retention and transfer test scores associated with the videos.

#### Time-on-task

The time that the students in both research designs spent on the learning task was automatically recorded by the system.

#### Cognitive load

According to Paas et al. (2003), the assessment of cognitive load involves mental load and mental effort. Mental load refers to the cognitive capacity demanded for carrying out a task. Mental effort is related to the cognitive capacity an individual invests in working on a task. The items measuring mental load and mental effort were adjusted from Hwang et al. (2013). Four items measured mental load, for instance, “The learning content in the instructional videos was generally difficult for me.” And four items assessed mental effort, for instance, “The instructional design of this task was difficult to follow and understand.” The Cronbach’s alpha values of mental load and mental effort were 0.90 and 0.92, respectively.

#### Learning agency

Learning agency was measured by five items, which were adapted from Jung et al. (2019). An illustrative item is “I made a plan to guide myself through the video-based learning.” The Cronbach’s alpha value of learning agency was 0.87.

#### Affective and cognitive engagement

Affective and cognitive engagement is related to students’ involvement of affection, effort, and cognition in learning (Bergdahl et al., 2020). It was assessed by seven items, which were developed from Bergdahl et al. (2020). The Cronbach’s alpha value was 0.93. A sample item is “I forget everything else around me when I was studying the instructional videos.”

#### Focus group

Focus group interviews were conducted at the end of this study to investigate in-depth the students’ attitudes and experiences of different learner control designs in video-based learning. The results of focus group were used to complement the quantitative findings of the comparisons between the two learner control conditions. Six students were randomly selected from FLC and HLC separately. General questions were asked, for instance, “How do you think of the instructional design?” and “How do you organize your learning?” Each focus group lasted about 30 min.

## Results

### Group comparison outcomes

To control for potential effects of gender on the intervention, we decided to perform MANCOVA instead of multiple rounds of *t*-test. The one-way MANCOVA indicated that there were significant differences between FLC and HLC on the combined dependent variables (e.g., time cost, overall score, learning agency) after controlling for gender, *F*(8, 76) = 3.79, *p* = 0.001 < 0.01, Wilks’ Λ = 0.72, partial η^2^ = 0.29, which implies that approximately 29% of the variance in the dependent variables was accounted for by the instructional designs.

As shown in Table 1, except for the score of easy instructional videos, mental load and effort, and affective and cognitive engagement, the remaining variables were statistically significant, with effect sizes varying from 0.07 (learning agency) to 0.24 (time cost).

**TABLE 1:** Descriptive statistics and pairwise comparisons.

Dependent variables	Intervention	Mean (SE)	*F*	*df*	Partial η^2^	Mean difference between FLC and HLC (*SE*)	Sig.	95% CI for difference
Time cost (Seconds)	FLC	5243.70 (146.76)	25.54	(1, 83)	0.24	1068.12* (211.35)	0.000	[647.76, 1488.49]
	HLC	4175.58 (150.25)						
Easy section score	FLC	17.88 (0.23)	0.84	(1, 83)	0.01	0.31 (0.34)	0.361	[–0.36, 0.98]
	HLC	17.57 (0.24)						
Difficult section score	FLC	15.43 (0.48)	7.65	(1, 83)	0.08	1.89* (0.69)	0.007	[0.53, 0.33]
	HLC	13.53 (0.49)						
Total retention test score	FLC	16.45 (0.34)	5.76	(1, 83)	0.07	1.18* (0.49)	0.019	[0.20, 2.15]
	HLC	15.27 (0.35)						
Total transfer test score	FLC	16.86 (0.31)	5.14	(1, 83)	0.06	1.03* (0.45)	0.026	[0.13, 1.93]
	HLC	15.84 (0.32)						
Overall score	FLC	33.31 (0.57)	7.20	(1, 83)	0.08	2.20* (0.82)	0.009	[0.57, 3.84]
	HLC	31.11 (0.58)						
Learning agency	FLC	4.39 (0.10)	6.06	(1, 83)	0.07	0.36* (0.15)	0.016	[0.07, 0.65]
	HLC	4.03 (0.10)						
Mental load	FLC	2.50 (0.15)	0.16	(1, 83)	0.002	0.08 (0.21)	0.695	[–0.34, 0.51]
	HLC	2.41 (0.15)						
Mental effort	FLC	2.51 (0.15)	1.20	(1, 83)	0.01	0.24 (0.22)	0.277	[–0.20, 0.69]
	HLC	2.27 (0.16)						
Affective and cognitive engagement	FLC	3.66 (0.13)	0.78	(1, 83)	0.01	0.17 (0.19)	0.381	[–0.21, 0.54]
	HLC	3.50 (0.13)						

*p < 0.05, adjusted for multiple comparisons. FLC, Full learner control; HLC, Hybrid learner control.

Specifically, the participants in FLC spent more time learning the instructional videos than those in HLC. They achieved significantly higher scores in the retention tests, transfer tests, and overall tests than those in HLC. They also demonstrated more learning agency in the learning process than the participants in HLC.

In particular, the finding of the participants’ performance in the videos of different difficulty levels was quite interesting. That is, the participants in FLC obtained similar scores in the easy section to but significantly higher scores in the difficult section than those in HLC. However, there were no significant differences between the two learner control designs in mental load, mental effort, and cognitive engagement.

### Hierarchical regression analysis

Separate hierarchical regression analyses were conducted to examine which factors predict students’ overall academic performance in the two designs. Durbin-Watson statistics for the participants’ overall scores in FLC and HLC were 2.07 and 1.94, respectively, implying no autocorrelation in the residuals for the two variables. Variance inflation factor (VIF) values ranged from 1.00 to 5.08, which were substantially lower than 10, suggesting no signs of multicollinearity in the models of the two designs.

In Model 1 of FLC, the students’ overall score was entered as the dependent variable with gender as a predictor. Model 1 was not statistically significant (see Table 2). In Model 2, mental load and mental effort were entered. The results indicate that Model 2 was statistically significant, *F*(3, 40) = 2.85, *p* = 0.050, adjusted *R*^2^ = 0.10. The *F-*value increased significantly in Model 2, Δ*F* = 3.30, *p* = *0.047* < 0.05. Mental load (β = –0.67, *p* = 0.014 < 0.05) and mental effort (β = 0.54, *p* = 0.042 < 0.05) were found to be significant after controlling gender. In Model 3, learning agency and affective and cognitive engagement were included, generating statistically significant model, *F*(5, 38) = 2.61, *p* = 0.040 < 0.05, adjusted *R*^2^ = 0.16. However, mental load (β = –0.48, *p* = 0.081 > 0.05) was no longer a significant predicator in Model 3 while the influence of mental effort (β = 0.70, *p* = 0.011 < 0.05) increased. Affective and cognitive engagement (β = 0.51, *p* = 0.037 < 0.05) became a significant predictor of students’ overall academic performance with gender, mental load, and mental effort taken into account.

**TABLE 2:** Hierarchical regression analysis for FLC with the total score as the independent variable (*N* = 44).

Variables	Model 1			Model 2			Model 3		
	*B*	*SE*	β	*B*	*SE*	β	*B*	*SE*	β
Constant	30.19	3.16		33.42	3.67		26.24	6.77	
Gender	1.64	1.67	0.15	0.63	1.65	0.06	0.31	1.64	0.03
Mental load				–2.45	0.95	–0.67*	–1.75	0.98	–0.48
Mental effort				1.89	0.90	0.54*	2.45	0.92	0.70*
Learning agency							–0.73	1.10	–0.12
Emotional and cognitive engagement							2.15	0.99	0.51*
*R* ^2^	0.02			0.16			0.26		
Adjusted *R*^2^	–0.001			0.10			0.16		
F	0.97			2.85*			2.61*		
Δ*R*^2^	0.02			0.14			0.10		
Δ*F*	0.97			3.30*			2.42		

*p < 0.05.

Hierarchical regression analysis was performed for HLC with the participants’ overall score as the dependent variable (see Table 3). The participants’ gender was entered into Model 1, which was found to be statistically significant, *F*(1, 40) = 4.34, *p* = 0.044 < 0.05, adjusted *R*^2^ = 0.08. Gender (β = –0.31, *p* = 0.044 < 0.05) was negatively associated with the participants’ overall score. Females (*M* = 30.48, *SD* = 3.55) tended to achieve lower scores than males (*M* = 33.09, *SD* = 3.62), *t*(40) = 2.08, *p* = 0.44. In Model 2, the inclusion of mental load and mental effort did not generate a statistically significant model. In Model 3, learning agency and affective and cognitive engagement were further included. There was a significant increase in the *F-*value, Δ*F* = 7.10, *p* = *0.003* < 0.01. Learning agency (β = 0.52, *p* = 0.003 < 0.01) was found to be a significant predictor of the participants’ overall academic performance with gender, mental load, and mental effort controlled.

**TABLE 3:** Hierarchical regression analysis for HLC with the total score as the independent variable (*N* = 42).

Variables	Model 1			Model 2			Model 3		
	*B*	*SE*	β	*B*	*SE*	β	*B*	*SE*	β
Constant	35.70	2.24		37.62	2.90		26.89	4.13	
Gender	–2.61	1.25	–0.31*	–2.80	1.30	–0.34*	–2.56	1.14	–0.31*
Mental load				–0.35	1.36	–0.09	–0.33	1.21	–0.08
Mental effort				–0.31	1.30	–0.08	–0.27	1.14	–0.07
Learning agency							2.64	0.82	0.52**
Emotional and cognitive engagement							–0.14	0.77	–0.03
*R* ^2^	0.10			0.12			0.37		
Adjusted *R*^2^	0.08			0.06			0.28		
F	4.34*			1.79			4.26**		
Δ*R*^2^	0.10			0.03			0.25		
Δ*F*	4.34			0.57			7.10**		

*p < 0.05; **p < 0.01.

## Discussion

The present study examined the functioning of the full learner control condition (FLC) and the hybrid learner control condition which involves both learner and instructor control (HLC) in video-based learning. In FLC, learners were allowed to freely jump around in a course consisting of a series of videos presented following an easy-to-complex principle. As such, FLC is similar to personalized learning where learners determine their learning path based on their situations and needs. In HLC, learners had to follow the presentation sequence of the course but could still control the display of each video and could watch any video freely once they completed the course. Pure instructor control where learners were not given any freedom at all was not examined in this study as it is rarely used in practical settings and is not helpful for conceptual learning (Schroeder et al., 2020). Overall, this study produced six findings.

First, the students in FLC achieved higher academic performance than those in HLC. In line with Moore (1993) and Jung et al. (2019), the full learner control in FLC greatly increased the students’ learning agency and may minimize the transactional distance between them and the course content as the students could freely choose what and how to learn, thereby increasing the interaction between them and learning resources and strengthening their understanding of the content knowledge. Consequently, the students in FLC obtained higher academic performance in both the retention and transfer tests than those in HLC. Contrastingly, the structured learning path set by the instructor in HLC might constrain the interaction between the students and the course content, thereby enlarging the transactional distance between the two and increasing their misunderstanding of the course content. As a result, the students in HLC may have a higher chance of making mistakes on the tests and achieving lower scores than their counterparts in FLC.

Second, the students in FLC scored higher than those in HLC in the difficult sections of video-based learning. But there was no significant difference between them in the easy section. This could be because the instructional design in FLC may enable the students to freely interact with the course content, promoting deep processing of information in the difficult video content in particular, and subsequently minimizing the transactional distance between the students and course content and enhancing their mastery of the content knowledge (Ekwunife-Orakwue and Teng, 2014; Li et al., 2021). In comparison, the hybrid learner control condition where the students had to follow the instructor-set learning path may be particularly effective for studying basic content and for developing low-order thinking skills (Jung et al., 2019). However, when the students achieve a certain proficiency, instructor-set learning path may obstruct the students from stretching their knowledge boundaries and improving higher-order thinking skills.

Third, the students in FLC spent significantly more time than those in HLC completing the video-based learning. This finding corroborated Tabbers and de Koeijer (2010), who found that giving learner control in multimedia learning could be at the cost of learning efficiency. It can be explained both by the instructor’s observation during the study and the post-study interview. The self-guided learning path in FLC was manifested in a variety of formats. There were students going through the tests first and then watching the videos with the tests in mind. There were also students doing the other way round. It seemed that every student had his/her distinct learning path and there was not a consistent and general pattern applying to most students. Many were seen constantly going back and forth in the instructional videos and tests. The interviewed students indicated that they watched certain segments of videos several times before moving to another one. As a result, the students in FLC spent quite a lot of time processing the video content, thereby likely enhancing their understanding of the video content (Fiorella and Mayer, 2018). In contrast, the entire learning process in HLC seemed to be smoother than that in FLC. They watched the videos and did the tests one by one. Many did not bother to replay the videos to check their mastery of the knowledge or check their answers to the tests. Some of the interviewed students stated a strong sense of complying with the instructor’s requirement, instead of feeling ownership of their study. This may explain why a number of students seemed to be in a rush to finish the task as quickly as possible as observed by the instructor. Consequently, the students in HLC might not have been able to meaningfully process each instructional video before moving on to the next.

Fourth, the insignificant difference in the affective and cognitive engagement in FLC and HLC was similar to Tabbers and de Koeijer (2010) who found that the availability of learner control did not increase students’ involvement with the learning task. The insignificant differences in mental load and effort between the two designs are congruent with Tabbers and de Koeijer (2010) and Merkt et al. (2018) who found that the variations of learner control were not necessarily related to cognitive load as the students might develop different strategies to cope with the transient flow of information from the videos.

Fifth, as for the hierarchical regression analysis of FLC, mental load showed a significant negative effect on the overall score while mental effort showed a positive effect in Model 2. But mental load became less important when affective and cognitive engagement was included in Model 3. As argued by Biard et al. (2018) and Schroeder et al. (2020), if students do not know how to direct video-based learning, they may choose an unsuitable learning path, which may incur considerable mental load on them and require substantial mental effort to process video materials. However, on the other hand, proper learner control can stimulate students’ interest and motivation in video-based learning, which can increase their affective and cognitive engagement and later mitigate the requirements that the video content imposes on their cognitive capacities (Scheiter and Gerjets, 2007; Biard et al., 2018).

And sixth, as for the hierarchical regression analysis of HLC, gender negatively predicted the overall score in Model 1. This was probably caused by the significant lower overall score of females (*M* = 30.48; *SE* = 0.64) than males (*M* = 33.09; *SE* = 1.09) in HLC, *t*(40) = 2.08, *p* = 0.04. Learning agency became a significant predictor of the overall score in Model 3. In line with Mayer (2005) and Fiorella et al. (2020), the hybrid mode involving both learner and instructor control might have made the students feel as if they were partnering with their instructor, thereby prompting agentic actions to actively master the video content.

### Contributions

This study has the following contributions. First, the research findings contribute to the literature about learner control, which is considered a viable instructional design for increasing learning, by revealing which kinds of learner control, under what conditions, are beneficial for students’ video-based learning.

Second, this study underscores the importance of different ways of student-content interaction in digital environments and provides empirical support for the effectiveness of transactional distance theory, cognitive load theory, and the cognitive theory of multimedia learning in jointly explaining how students process information in instructional videos to optimize their learning.

Third, contrary to many prior studies that compared learner control with system control in a single instructional video, this study investigated two learner control designs (the full and hybrid learner control) in a mini course involving multiple videos, which are more common in practice. As such, the findings of different learner control designs can be more generalizable to authentic educational practice.

### Implications

The results of this study can inform the design and implementation of a variety of video-based learning such as flipped classrooms and online courses. First, allowing students full control over the pacing and sequencing of instructional videos can facilitate the students to actively process video content, thereby leading to high learning performance. But if there is a constraint of time for students, it is advised to adopt the hybrid learner control design where the students follow the instructor-set path but still enjoy a certain amount of control over their learning.

Second, as for the course content of low difficulty levels, either the full learner control design or the hybrid learner control design is fine. However, given that the hybrid learner control design is time-saving, it would be better to adopt it for this type of video course.

Third, with regard to the course content of high difficulty levels, it is suggested to apply the full learner control design so as to enable students to direct their learning based on their capacities and needs.

Fourth, as MOOCs, which are primarily comprised of videos (Stöhr et al., 2019), increasingly become a strong force in higher education and many MOOC platforms actually give learners full control over their learning sequences (Lundqvist and Warburton, 2019), MOOC developers can use the comparison between the full learner control and the hybrid learner control in this study to determine the strengths and weaknesses of both conditions so as to optimize the online video-based learning design (Ginda et al., 2019).

### Limitations and future research

Some cautions should be borne in mind when interpreting the research findings. First, the uneven number of genders in both instructional designs, which was caused by convenience sampling, may present a constraint to the generalizability of the research findings. Future studies are suggested to validate the findings using a more balanced sample.

Second, the participants of this study were university students already enrolled in higher education with a narrow age range. However, online courses such as MOOCs attract a much broader age range and more diversified backgrounds of population groups. Thus, future studies could examine if and how different design conditions work for learners from different demographic backgrounds.

Third, the findings of this study shall apply to conceptual learning through instructional videos, instead of procedural learning. However, researchers may consider testing these findings in procedural learning to examine their generalizability and enrich the findings related to the learner control designs for different purposes.

Fourth, it is unclear whether and how the extra time-on-task in FLC could produce higher performance than HLC. To identify whether it is simply a matter of more time-on-task, future research is suggested to test another instructional design that allows participants to have more time in learning instructional videos without letting them control the sequencing and pacing of the instructional videos.

## Conclusion

Overall, the full learner control condition in FLC is analogous to a buffet where individuals take what they want; whilst, the hybrid learner control condition in HLC is analogous to a preset pack where individuals have to follow nutritionists’ suggestions so as to gain necessary nutrients to grow up. Both the buffet and the preset pack have merits in themselves and it is at the discretion of learners and instructors to choose which one to adopt.

## Data availability statement

The raw data supporting the conclusions of this article will be made available by the authors, without undue reservation.

## Ethics statement

The studies involving human participants were reviewed and approved by the Institutional Review Board of Qingdao University. The patients/participants provided their written informed consent to participate in this study.

## Author contributions

LL designed the study, helped with the data collection, and analyzed the data. XW designed the study, analyzed the data, and wrote the manuscript. MW helped with the data analysis and edited the manuscript. All authors contributed to the article and approved the submitted version.
