# Supplementary material for: I determine my learning path, or not? A study of different learner control conditions in online video-based learning
Source: Front Psychol. 2022 Sep 8;13:973758. doi: 10.3389/fpsyg.2022.973758 (PMC9496864; doi:10.3389/fpsyg.2022.973758)
Supplement: Supplementary file 1 [file Data_Sheet_1.docx]

Supplementary Material

**Appendix A. Survey items**

| **Constructs** | **Items** | **Sources** |
| --- | --- | --- |
| Learning agency | (LA1) I determined the pacing of this video-based learning. | Jung et al. (2019) |
|  | (LA2) I made a plan to guide myself through this video-based learning. |  |
|  | (LA3) I did my best to mobilize my attention to learn the video content. |  |
|  | (LA4) I determined the amount of time and effort invested in learning every video. |  |
|  | (LA5) I was active in participating in this video-based learning. |  |
| Mental load | (ML1) It was troublesome for me to answer the questions in the video courses. | Hwang et al. (2013) |
|  | (ML2) The learning content in the videos was generally difficult for me. |  |
|  | (ML3) I felt frustrated with answering the questions in the video courses. |  |
|  | (ML4) I did not have enough time to comprehend well the video content. |  |
| Mental effort | (ME1) The instructional design of this video-based learning caused me a lot of mental effort. |  |
|  | (ME2) I needed to invest substantial efforts in coping with the requirements of this video-based learning. |  |
|  | (ME3) I needed to invest substantial efforts to attain a high score. |  |
|  | (ME4) The instructional design of this video-based learning was difficult to follow and understand. |  |
| Affective & cognitive engagement | (ENG1) I was enthusiastic about this instructional design. | Bergdahl et al. (2020) |
|  | (ENG2) I found learning statistics through this way was full of meaning and purpose. |  |
|  | (ENG3) I forgot everything else around me when I was studying the videos. |  |
|  | (ENG4) I got carried away when I was studying the videos. |  |
|  | (ENG5) I was immersed in this video-based learning. |  |
|  | (ENG6) I felt happy when I was studying the videos intensively. |  |
|  | (ENG7) I could continue to study statistics through this way for a long time. |  |

**Appendix B. Sample retention and transfer test items**

(**Sample retention test questions for Dispersion**). Which statement in the following is wrong? (correct answer: B)

1. Mean, median, and mean lose a lot of information when describing the data situation.
2. The mean does not reflect whether the numbers in the set tend to be close to the mean or far away from the mean.
3. The overall variance can measure the degree of data fluctuation; the lower the value, the greater the fluctuation.
4. The overall variance is the average of the sum of squares of the difference between each data and the overall mean.

(**Sample transfer test questions for Dispersion**). The table below shows the average number of rings and variance obtained by four shooters A, B, C, and D in a tryout. The best candidate for the final is: (correct answer: C)

|  | A | B | C | D |
| --- | --- | --- | --- | --- |
| Average | 8 | 9 | 9 | 8 |
| VAriance | 5.7 | 6.2 | 5.7 | 6.4 |

(**Sample retention test item for Probability density function**) The following figure shows the probability density function of the rainfall in a city. The abscissa is the number of inches of rainfall. Which statement in the following is wrong? (correct answer: B)

1. The abscissa is a random variable, and the ordinate is a probability density function.
2. When the random variable is 2, the probability density function is 0.5. Therefore, the probability that the rainfall reaching 2 inches *p*{*x*=1} is 0.5.
3. The probability that the rainfall is exactly 1 inch *p*{*x*=1} is very small.
4. The probability *p*{1.9<*x*<2.1} that the rainfall is in the interval of 1.9-2.1 is obtained by calculating the area under the curve in the interval of 1.9-2.1 on the abscissa.

(**Sample transfer test item for Probability density function**) Suppose the distribution function of the continuous random variable x is $f\left( x \right)=\left\{ \begin{aligned} kx, &0\leq x\leq1 \\ 0, &x<0 or x＞1 \end{aligned} \right.$, find *p*（*x*≤0.5）(correct answer: C)

1. *p*（*x*≤0.5）= 0.05
2. *p*（*x*≤0.5）= 0.15
3. *p*（*x*≤0.5）= 0.25
4. *p*（*x*≤0.5）=0.35
